# Reassessing China’s Regional Modernization Based on a Grey-Based Evaluation Framework and Spatial Disparity Analysis

**DOI:** 10.3390/e28010117

**Published:** 2026-01-19

**Authors:** Wenhao Zhou, Hongxi Lin, Zhiwei Zhang, Siyu Lin

**Affiliations:** 1 Business School, Putian University, Putian 351100, China; wenhaoz2021@stu.hqu.edu.cn (W.Z.);; 2 School of Business Administration, Capital University of Economics and Business, Beijing 100070, China

**Keywords:** Chinese modernization, sustainable development, regional disparities, grey relational analysis, multi-criteria evaluation

## Abstract

Understanding regional disparities in Chinese modernization is essential for achieving coordinated and sustainable development. This study develops a multi-dimensional evaluation framework, integrating grey relational analysis, entropy weighting, and TOPSIS to assess provincial modernization across China from 2018 to 2023. The framework operationalizes Chinese-style modernization through five dimensions: population quality, economic strength, social development, ecological sustainability, innovation and governance, capturing both material and institutional aspects of development. Using K-Means clustering, kernel density estimation, and convergence analysis, the study examines spatial and temporal patterns of modernization. Results reveal pronounced regional heterogeneity: eastern provinces lead in overall modernization but display internal volatility, central provinces exhibit gradual convergence, and western provinces face widening disparities. Intra-regional analysis highlights uneven development even within geographic clusters, reflecting differential access to resources, governance capacity, and innovation infrastructure. These findings are interpreted through modernization theory, linking observed patterns to governance models, regional development trajectories, and policy coordination. The proposed framework offers a rigorous, data-driven tool for monitoring modernization progress, diagnosing regional bottlenecks, and informing targeted policy interventions. This study demonstrates the methodological value of integrating grey system theory with multi-criteria decision-making and clustering analysis, providing both theoretical insights and practical guidance for advancing balanced and sustainable Chinese-style modernization.

## 1. Introduction

### 1.1. Background

As the world’s largest developing country, China has made remarkable progress in its pursuit of modernization. Yet this process remains complex, uneven, and far from complete. Rather than a purely economic transformation, China’s modernization represents a multidimensional and evolving process involving economic restructuring, social development, ecological sustainability, governance capacity, and innovation [1,2]. Distinct from Western modernization paradigms that have historically prioritised industrialisation and consumption-driven growth, Chinese modernization emphasises balanced development, ecological civilisation, common prosperity, and long-term societal well-being [3].

Despite decades of rapid growth, China continues to face persistent structural challenges, including pronounced regional disparities between coastal and inland areas, environmental degradation, demographic aging, and urban-rural disparities [4,5,6]. In response, the concept of “Chinese-style modernization” has been formally articulated as a development pathway tailored to national conditions, explicitly highlighting inclusiveness, sustainability, and governance effectiveness. While this concept has gained increasing attention in both policy discourse and academic research, a critical question remains insufficiently addressed: how can the level and quality of modernization be scientifically and systematically evaluated across China’s highly heterogeneous regions?

Given China’s vast territorial scale and strong spatial differentiation, national-level indicators alone are inadequate for capturing the uneven progress of modernization. Provincial-level assessment is essential for identifying regional bottlenecks, monitoring dynamic changes, and supporting differentiated policy interventions. Such an assessment framework must move beyond narrow economic indicators to incorporate broader social, environmental, governance, and innovation-related dimensions that underpin long-term development and population well-being. However, existing modernization studies often rely on single-dimensional economic metrics or qualitative assessments, limiting their usefulness for evidence-based regional policymaking [1,2]. Moreover, many quantitative evaluations insufficiently account for spatial disparity, sustainability considerations, and the inherent uncertainty and complexity of modernization processes.

To address these limitations, this study proposes an integrated and data-driven evaluation framework that combines the grey relational model, entropy weight method, and the Technique for Order Preference by Similarity to Ideal Solution (TOPSIS). This composite approach is designed to capture the multidimensional and “grey” nature of regional modernization, characterised by incomplete information, non-linear interactions, and heterogeneous development trajectories. By integrating objective weighting with relational analysis and ideal-solution-based ranking, the framework aims to provide a more robust and interpretable measurement of modernization performance across regions. Using provincial data for China’s 31 provinces from 2018 to 2023, this study not only assesses the relative level of modernization but also examines its spatial distribution and temporal dynamics. In addition, clustering analysis and kernel density estimation are employed to explore patterns of convergence, divergence, and intra-regional heterogeneity among eastern, central, and western regions. These analyses help reveal how modernization pathways differ across space and over time, offering insights into the structural drivers of regional inequality.

The objectives of this study are threefold. First, it seeks to construct a scientifically grounded, multidimensional, and sustainability-oriented indicator system for evaluating regional modernization in China. Second, it aims to identify spatial disparities and temporal evolution in modernization performance across provinces and major regions. Third, it contributes methodologically by integrating grey system theory with entropy-based weighting, TOPSIS ranking, and spatial analysis to enhance the robustness and interpretability of modernization assessment.

By linking modernization evaluation to sustainability and regional equity, this study contributes to a deeper understanding of China’s differentiated modernization trajectory. The proposed framework offers a practical diagnostic tool for policymakers and regional planners to identify development bottlenecks, design targeted interventions, and promote more balanced and health-oriented modernization pathways. Beyond the Chinese context, the study also provides methodological insights relevant to other large and spatially diverse developing economies.

### 1.2. Literature Review

A clear understanding of the conceptual foundations and distinctive features of Chinese modernization is crucial for its accurate measurement. Chinese modernization has been characterised as a process encompassing the modernization of a large population, the pursuit of common prosperity, the coordination of material and cultural–ethical advancement, harmony between humanity and nature, and peaceful development [7]. Unlike Western modernization paths shaped by early industrialisation and neoliberal expansion, Chinese modernization is rooted in the country’s specific historical conditions, institutional arrangements, and developmental stage, and is increasingly viewed as offering alternative insights into global development trajectories [8].

The sheer scale of China’s population presents unique challenges for modernization. Achieving modernization for over 1.4 billion people implies a prolonged and uneven process, particularly when combined with strong regional heterogeneity [7]. Within this context, common prosperity has emerged as a defining goal, reflecting a commitment to inclusive development rather than polarised wealth accumulation [9]. At the same time, Chinese modernization emphasises the coordinated advancement of material conditions and cultural–ethical life, recognising that neither material abundance nor spiritual development alone is sufficient for socialist modernization [9]. Rapid economic growth since the reform and opening-up period has also generated significant environmental pressures, including pollution, ecosystem degradation, and biodiversity loss [10]. In response, sustainability has become a central pillar of Chinese modernization, embodied in concepts such as high-quality development and ecological civilisation. These principles underscore the need to reconcile economic progress with environmental protection and long-term societal resilience [7]. Governance capacity and innovation further shape modernization outcomes by influencing policy implementation efficiency, institutional quality, and the transition toward knowledge-driven development.

Against this theoretical backdrop, scholars have increasingly attempted to evaluate Chinese modernization through indicator-based approaches. Existing studies have developed composite indices focusing on dimensions such as common prosperity [11], urbanization, rural development [12], and provincial modernization [13,14,15,16]. These efforts have enriched the empirical understanding of modernization but often rely on conventional evaluation methods such as entropy weighting, fuzzy comprehensive evaluation, principal component analysis, or simple composite indices. While useful, these methods exhibit notable limitations, including sensitivity to data structure, loss of information in dimensionality reduction, or excessive subjectivity in weighting schemes.

More importantly, modernization in China is a dynamic and regionally differentiated process characterised by uncertainty, non-linearity, and evolving policy priorities. Traditional evaluation approaches often struggle to capture these features, particularly when assessing interprovincial comparability and temporal evolution under incomplete information. As a result, there remains a need for an evaluation framework that is theoretically aligned with the multidimensional connotation of Chinese modernization, empirically robust to regional heterogeneity, and capable of supporting spatial and temporal analysis.

To meet this need, this study develops an integrated GR-EWT-TOPSIS framework grounded in the core dimensions of Chinese modernization. By combining grey relational analysis with entropy-based weighting and ideal-solution ranking, and by incorporating clustering and distributional analysis, the proposed approach seeks to overcome the limitations of traditional methods and provide a more nuanced understanding of regional modernization patterns. The empirical results offer evidence to support differentiated regional strategies, improved governance, and more sustainable and equitable development pathways.

The remainder of this paper is organized as follows: Section 2 presents the conceptual rationale and methodological framework. Section 3 introduces the indicator system and reports the empirical results. Section 4 discusses the main findings in relation to regional modernization theory and policy implications. Section 5 concludes with key insights, limitations, and future research.

### 1.3. Conceptual Rationale for the Integrated Framework

Assessing regional modernization in China presents a complex analytical challenge due to the multidimensional nature of modernization, pronounced regional heterogeneity, and the presence of uncertainty and non-linear interactions among development factors. Modernization is not a single-dimensional or linear process but rather a composite outcome shaped by economic capacity, social development, ecological sustainability, governance effectiveness, and innovation. These dimensions interact dynamically across regions that differ markedly in development stage, institutional capacity, and resource endowments. As a result, conventional evaluation approaches that rely on single indicators or static composite indices are often insufficient for capturing the full complexity of China’s regional modernization process.

Existing quantitative studies commonly employ methods such as entropy weighting, principal component analysis, or simple composite indices. While these approaches provide useful benchmarks, each exhibits inherent limitations when applied to the assessment of Chinese modernization. Entropy-based methods objectively assign weights based on data dispersion but may overlook relational information among indicators and regions. Principal component analysis reduces dimensionality but can obscure the original meaning of indicators and lead to information loss in highly multidimensional systems. Ideal-solution-based methods such as TOPSIS offer intuitive ranking outcomes but are sensitive to weighting schemes and data structure, potentially affecting result stability. When used in isolation, these methods struggle to simultaneously address uncertainty, multidimensional comparability, and interpretability.

Grey system theory provides a valuable analytical foundation for this context. Grey relational analysis is particularly well suited to systems characterised by incomplete information, limited samples, and uncertain relationships—features that are intrinsic to regional modernization evaluation. By focusing on the degree of similarity in development trends rather than absolute values, grey relational analysis captures the relative coordination among multiple dimensions of modernization across regions. However, grey relational analysis alone does not generate a comprehensive performance ranking or account for the differential informational contribution of indicators.

To address these limitations, this study integrates grey relational analysis with entropy-based weighting and the TOPSIS method. Entropy weighting is employed to objectively determine indicator weights based on their information content, thereby reducing subjectivity and enhancing the robustness of the evaluation. TOPSIS is then used to translate multidimensional indicator performance into an interpretable modernization score by measuring each region’s relative proximity to an ideal modernization state. Within this integrated framework, grey relational analysis enhances sensitivity to developmental patterns under uncertainty, entropy weighting ensures data-driven importance assignment, and TOPSIS provides clear and policy-relevant ranking outcomes. The integration of these methods is thus complementary rather than arbitrary.

Importantly, the GRM–EWT–TOPSIS framework enables analytical insights that are difficult to obtain through traditional approaches. It allows for stable interprovincial comparisons over time, facilitates the identification of dimension-specific development bottlenecks, and supports spatial and temporal analysis of modernization trajectories. When combined with clustering analysis and distributional techniques, the framework further reveals patterns of convergence, divergence, and intra-regional heterogeneity, offering a more nuanced understanding of China’s differentiated modernization pathways.

Grounded in the core dimensions of Chinese modernization and aligned with sustainability-oriented development goals, the proposed framework provides a transparent and flexible tool for monitoring regional modernization performance. By explicitly linking methodological choices to the conceptual characteristics of modernization, this approach strengthens the theoretical coherence and policy relevance of the empirical analysis, making it particularly suitable for evaluating complex, large-scale, and regionally diverse development processes such as Chin’s modernization.

## 2. Methodology

### 2.1. Grey Relational Analysis Method

Assume that a normalized evaluation unit is $X=\left\{X_{1},X_{2},\cdots ,X_{m}\right\}$, with each unit having *n* indicators and *m* evaluation objects, the evaluation index matrix for each individual year *t* can be represented as

(1) $$X={\left|\begin{array}{cccccc} x_{11}^{t} & x_{12}^{t} & \cdots & x_{1j}^{t} & \cdots & x_{1n}^{t}\\ x_{21}^{t} & x_{22}^{t} & \cdots & x_{2j}^{t} & \cdots & x_{2n}^{t}\\ \vdots & \vdots & \ddots & \vdots & \ddots & \vdots \\ x_{i1}^{t} & x_{i2}^{t} & \cdots & x_{ij}^{t} & \cdots & x_{ij}^{t}\\ \vdots & \vdots & \ddots & \vdots & \ddots & \vdots \\ x_{m1}^{t} & x_{m2}^{t} & \cdots & x_{mj}^{t} & \cdots & x_{mn}^{t} \end{array}\right|}_{m\times n}$$

GRA (Grey Relational Analysis, GRA), is a crucial method of system analysis that describes the strength of relationships between factors by measuring the dynamic development trends within the system. Unlike traditional mathematical statistical methods, GRA does not require a specific size or distribution pattern for the sample data. It studies the comparative process of dynamic changes between indicator factors, so there is no inconsistency between qualitative analysis and quantitative calculation results. The larger the result value of GRA, the greater the degree of relationship between different indicator factors.

The most widely applied and deeply researched model is the point grey relational model, also known as the proximity grey relational degree, first proposed by Professor Deng in 1982 [17]. Its basic calculation method is as follows:

(2) $$\gamma \left(X_{0},X_{j}\right)=\frac{1}{t}\sum_{k=1}^{t}\frac{\underset{j}{\min}\;\underset{k}{\min}\left|x_{0}\left(k\right)-x_{j}\left(k\right)\right|+\rho \;\underset{j}{\max}\;\underset{k}{\max}\left|x_{0}\left(k\right)-x_{j}\left(k\right)\right|}{\left|x_{0}\left(k\right)-x_{j}\left(k\right)\right|+\rho \;\underset{j}{\max}\;\underset{k}{\max}\left|x_{0}\left(k\right)-x_{j}\left(k\right)\right|},k=1,2,\cdots ,t$$

In Equation (2), *x*_0_*(k)* and *x_i_(k)* represent the value to be compared and the observed value of the *j*-th indicator factor of sample *X*_0_ at time *t*, respectively, *ρ* is the resolution coefficient, generally taken as 0.5. Then, Equation (2) is the gray relational proximity between factor sequence *X_j_* and comparison sequence. The closer the relational degree value is to 1, the more similar the dynamic development degree between the factor sequence and the comparison sequence.

Grey relational analysis quantifies the closeness between the observed provincial modernization profiles and an idealized reference profile. Conceptually, this captures how well each province aligns with the target of Chinese-style modernization across multiple dimensions. Provinces with higher grey relational coefficients are interpreted as being closer to achieving balanced and sustainable modernization. From a policy perspective, this allows decision-makers to identify regions that require additional support or strategic interventions, providing a structured approach to monitor progress toward equitable and coordinated development.

TOPSIS (Technique for Order Preference by Similarity to Ideal Solution, TOPSIS) method, is a multi-criteria decision analysis approach [18]. Its core idea is to calculate the Euclidean distance between the target solution and an ideal solution, considering the solution with the shortest distance as the optimal one [19]. However, the TOPSIS method can only reflect the positional relationship between data curves and cannot capture the changing trend of data. The basic calculation method of TOPSIS method is as follows:

Step one: Determine the Positive ideal solution and Negative ideal solution for each indicator in the year *t*.

(3) $$X^{+}=\left\{x_{1}^{+},x_{2}^{+},\cdots ,x_{n}^{+}\right\}$$

(4) $$X^{-}=\left\{x_{1}^{-},x_{2}^{-},\cdots ,x_{n}^{-}\right\}$$

In particularly, the Positive ideal solution is the ideal optimal value of each index, and the Negative ideal solution is the set of the worst values of each index.

Step two: Calculate the sum of Euclidean distances for all indicators of evaluation object *i* from both the Positive ideal solution and the Negative ideal solution.

(5) $$D_{i}^{+}={\left({\sum_{j=1}^{n}\left|x_{ij}-x_{j}^{+}\right|}^{n}\right)}^{\frac{1}{n}},j=1,2,\cdots ,n,i=1,2,\cdots ,m$$

(6) $$D_{i}^{-}={\left({\sum_{j=1}^{n}\left|x_{ij}-x_{j}^{-}\right|}^{n}\right)}^{\frac{1}{n}},j=1,2,\cdots ,n,i=1,2,\cdots ,m$$

Step three: Calculate the Similarity to the ideal solution:

(7) $$C_{i}=\frac{D_{i}^{+}}{D_{i}^{+}+D_{i}^{-}},i=1,2,\cdots ,m$$

Step four: Rank the solutions according to *C_i_*.

Clustering is an unsupervised learning method that groups data based on inherent spatial distribution, without predefined rules, aiming for minimal within-group differences and maximal between-group differences [20]. K-Means, a centroid-based algorithm, partitions data into *k* clusters by iteratively assigning points to the nearest center and updating cluster centroids until convergence. Its simplicity, efficiency, and interpretability make it well-suited for evaluating regional modernization patterns. Conceptually, the K-Means clustering captures natural groupings of provinces based on their overall modernization profiles. Provinces assigned to the same cluster share similar developmental characteristics, while differences between clusters highlight structural disparities across regions.

**Figure 1:**
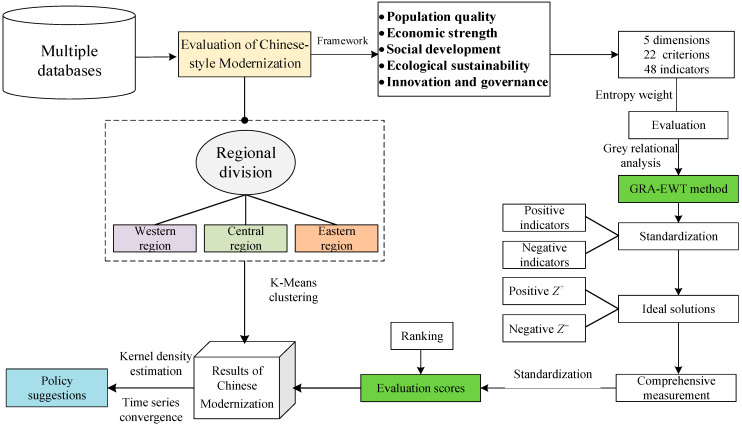
Modeling process of this study.

The main calculation process is as follows: Suppose a normalized dataset $\left\{x_{1},x_{2},\cdots ,x_{m-1},x_{m}\right\}$, the process of K-Means starts by randomly selecting *k* distinct cluster centers $\left\{c_{1},c_{2},\cdots ,c_{k}\right\}$ from the samples. Then calculate the Euclidean distances of the *m* different samples to these cluster centers:

(8) $$dist\left(x_{i},c_{j}\right)=\sqrt{\sum_{i=1}^{m}{\left(x_{i}-{\bar{c}}_{j}\right)}^{2}}$$

where ${\bar{c}}_{j}$ is the cluster center of the *j*-th cluster, which also represents the mean of the samples within the cluster across all dimensions. Therefore, the ultimate goal of the K-Means algorithm is to assign all samples to *k* different cluster centers, achieving the smallest possible intra-cluster variance and the largest possible inter-cluster variance. The implementation process is as shown in Figure 1.

### 2.2. Entropy Weight Method

The entropy weighting method is an objective way of assigning weights, determining weights based on the differences in the orderliness of information contained in each indicator. The greater the dispersion of an indicator, the larger the amount of information it contains, and consequently, the greater its impact or weight on the evaluation result [21]. This study incorporates a time variable into the traditional entropy weighting method, comprehensively considering the weight distribution for all sample data on each evaluation indicator, thereby obtaining more objective evaluation and analysis results. The specific calculation is as follows:

Step one: Determine the initial indicators. For *m* samples, *n* evaluation indicators, and *t* years, then $x_{ik}^{\left(j\right)}$ represents the value of the *j*-th indicator of the *i*-th sample in the *k* year.

Step two: Homogenization of heterogeneous indicators. Since the dimensions and units of each indicator are not uniform, it is necessary to standardize before calculating the evaluation results to solve the homogenization issue of different indicators. For benefit-type indicators, the standardization method is

(9) $${y_{ik}}^{\left(j\right)}=\frac{{x_{ik}}^{\left(j\right)}-\underset{i}{\min}{x_{ik}}^{\left(j\right)}}{\underset{i}{\max}{x_{ik}}^{\left(j\right)}-\underset{i}{\min}{x_{ik}}^{\left(j\right)}}$$

For cost-type indicators, the standardization method is:

(10) $${y_{ik}}^{\left(j\right)}=\frac{\underset{i}{\max}{x_{ik}}^{\left(j\right)}-{x_{ik}}^{\left(j\right)}}{\underset{i}{\max}{x_{ik}}^{\left(j\right)}-\underset{i}{\min}{x_{ik}}^{\left(j\right)}}$$

Step three: Calculate the proportion of the *i*-th sample in all samples for the *j*-th indicator:

(11) $${p_{ik}}^{\left(j\right)}=\frac{{y_{ik}}^{\left(j\right)}}{\sum_{i=1}^{m}{y_{ik}}^{\left(j\right)}}$$

Step four: Calculate the information entropy value of the *j*-th indicator in the *k*-th year:

(12) $$e_{jk}=-\frac{1}{\ln\left(n\right)}\sum_{i=1}^{m}{p_{ik}}^{\left(j\right)}\ln({p_{ik}}^{\left(j\right)}),\;j=1,2,\cdots ,n$$

Specifically, when ${p_{ik}}^{\left(j\right)}=0$, ${p_{ik}}^{\left(j\right)}\ln({p_{ik}}^{\left(j\right)})=0$.

Step five: Calculate information redundancy:

(13) $$d_{jk}=1-e_{jk},\;j=1,2,\cdots ,n$$

Step six: Calculate the weights of each indicator in a specific year *k*:

(14) $$w_{jk}=\frac{d_{jk}}{\sum_{j=1}^{n}d_{jk}}$$

Step seven: Average the weights of indicator *j* across different years to obtain the final indicator weight in the comprehensive evaluation system:

(15) $$w_{j}=\frac{1}{t}\sum_{k=1}^{t}w_{jk}$$

The entropy weight method objectively determines the contribution of each indicator to the overall modernization assessment based on the degree of variation among provinces. In conceptual terms, indicators with greater variation carry more information about regional differences and thus receive higher weights. This ensures that the evaluation emphasizes dimensions that most strongly distinguish development performance.

### 2.3. Grey Relation Model Based on Entropy Weight and TOPSIS

Through an in-depth study of the grey relational degree model, it has been found that the proximity grey relational degree overlooks the issue of differing contributions of various indicator factors to system changes during the dynamic development process, that is, it does not fully consider the weight distribution among various indicators [22]. Although some scholars have discussed the issue of weighting, a unified method for determining weights has not yet been established [23]. This paper first calculates the degree of variation in each indicator factor based on the entropy weight method, revises the weights of each indicator, and combines the basic principle of approaching the ideal solution in TOPSIS [24] with grey relational analysis to construct a grey relation model based on entropy weight and TOPSIS. This model overcomes the deficiency of the TOPSIS method in not reflecting the dynamic changes in data series, and simultaneously integrates the respective advantages of the TOPSIS method and grey relational analysis. The calculation method is as follows:

Step one: Calculate the weights of each indicator according to Equations (9)–(15) and obtain the indicator weight set $w_{j}=\left\{w_{1},w_{2},\cdots ,w_{n}\right\}$.

Step two: Determine the Positive ideal solution $X^{+}=\left\{x_{1}^{+},x_{2}^{+},\cdots ,x_{n}^{+}\right\}$ and Negative ideal solution $X^{-}=\left\{x_{1}^{-},x_{2}^{-},\cdots ,x_{n}^{-}\right\}$ according to Equation (4).

Step three: Calculate the score of the *i*-th evaluation object:

(16) $$CM_{i}=\frac{\gamma \left(U_{i},X^{+}\right)}{\gamma \left(U_{i},X^{+}\right)+\gamma \left(U_{i},X^{-}\right)},i=1,2,\cdots ,m$$

Herein, *U_i_* represents the indicator vector of the *i*-th object. According to Equation (2), $\gamma \left(U_{i},X^{+}\right)$ denotes the proximity grey relational degree between this object’s indicator vector and the Positive ideal state, and $\gamma \left(U_{i},X^{-}\right)$ represents the proximity grey relational degree between this object’s indicator vector and the Negative ideal state. Therefore, according to Equation (7), the final evaluation result for the *i*-th object is obtained. The closer its value is to 1, the better the evaluation result, indicating a state closer to the ideal. In this paper, it signifies a higher level of Chinese modernization.

For the GRM-EWT evaluation results, the evaluation grades of different samples vary. The evaluation grades are divided into intervals according to the final relational evaluation results, as shown in Table 1. Here, Grade I indicates that the region’s level of Chinese modernization is high, and its development model closely aligns with the connotation of Chinese modernization, belonging to a stage of high-quality development; Grade IV, on the other hand, indicates a lower level of Chinese modernization, with internal developmental imbalances and deviations from China’s current development model, necessitating timely adjustments by the relevant government departments.

Integrating grey relational analysis with entropy-weighted TOPSIS allows for a comprehensive and comparative ranking of provincial modernization levels. Conceptually, the resulting “modernization score” reflects both the closeness to the ideal development path and the relative significance of each indicator. The classification into Level I, II, and III can thus be interpreted as distinct stages of modernization progress, rather than merely numerical rankings. This provides policymakers with actionable insights, identifying not only the provinces that are leading in modernization but also those requiring strategic interventions in areas such as governance, innovation, or ecological management to achieve more balanced national development.

## 3. Evaluation System and Data Analysis Results

### 3.1. Construction of the Indicator System

**Figure 2:**
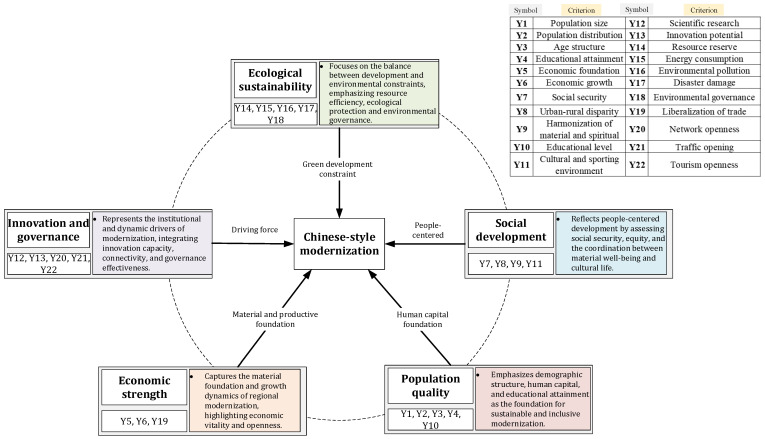
The dimensions of Chinese-style modernization.

Based on the theoretical connotation of Chinese-style modernization and the principles of scientific validity, data availability, and regional comparability, this study constructs a comprehensive evaluation indicator system, as shown in Figure 2, consisting of five core dimensions: population quality, economic strength, social development, ecological sustainability, and innovation and governance. Each indicator is uniquely assigned to one dimension to ensure conceptual clarity and computational consistency. The system is designed to capture not only the material foundations of modernization, but also its people-centered orientation, ecological constraints, and innovation-driven development logic. The relevant data were collected from multiple databases such as the China Statistical Yearbook (http://www.stats.gov.cn/sj/ndsj/, accessed on 6 November 2025), China Ecological Environment Status Bulletin (https://www.mee.gov.cn/hjzl/, accessed on 6 November 2025), China Health and Wellness Statistical Yearbook, China Environmental Statistics Yearbook.

Population quality constitutes a foundational dimension of Chinese-style modernization, reflecting the shift from demographic scale advantages toward human-capital-driven development. Unlike traditional modernization frameworks that emphasize population size alone, Chinese modernization increasingly stresses population structure, educational attainment, and the sustainability of demographic development. This dimension integrates indicators of population size (Y1), population distribution (Y2), age structure (Y3), educational attainment (Y4), and educational level (Y10). Together, these indicators capture both quantitative and qualitative aspects of population development, highlighting how balanced demographic structures and higher levels of education support long-term economic vitality, social stability, and innovation capacity across regions.

Economic strength represents the material foundation of modernization and remains a core pillar of Chinese-style modernization. However, rather than prioritizing growth at all costs, contemporary Chinese modernization emphasizes high-quality, efficient, and open economic development. This dimension focuses on the basic economic capacity and growth momentum of regions, as well as their degree of integration into broader domestic and international markets. Economic foundation (Y5) and economic growth (Y6) capture the scale and dynamism of regional economies, while trade liberalization (Y19) reflects openness and external economic engagement. These indicators jointly describe how regions generate material wealth while adapting to an increasingly open and competitive development environment.

Social development embodies the people-centered orientation of Chinese-style modernization and its commitment to common prosperity. Beyond economic performance, modernization in the Chinese context seeks to translate development outcomes into tangible improvements in social welfare, equity, and cultural life [25]. This dimension evaluates the inclusiveness and quality of social development through indicators of social security coverage (Y7), urban–rural disparities (Y8), the harmonization of material and spiritual life (Y9), and the cultural and sporting environment (Y11). Together, these indicators reflect the extent to which regional development enhances social protection, reduces structural inequalities, and fosters a supportive social and cultural environment, thereby strengthening social cohesion and development resilience.

Ecological sustainability is a defining feature of Chinese-style modernization and reflects the strategic vision of achieving harmony between humanity and nature. Recognizing the environmental pressures generated by rapid industrialization, China’s modernization path places strong emphasis on green development, resource efficiency, and effective environmental governance [26,27]. This dimension assesses regional performance in balancing economic and social development with ecological constraints. Indicators including resource reserves (Y14), energy consumption (Y15), environmental pollution (Y16), disaster damage (Y17), and environmental governance (Y18) jointly capture both environmental pressures and governance responses. By integrating ecological endowments, risks, and regulatory capacity, this dimension highlights regional differences in sustainable and resilient modernization pathways.

Innovation and governance represent the dynamic driving forces and institutional foundations of Chinese-style modernization. Innovation is not treated as an isolated technological process, but as an outcome shaped by openness, infrastructure connectivity, and governance capacity [28]. This dimension therefore integrates scientific research (Y12) and innovation potential (Y13) with indicators reflecting digital and physical openness, including network openness (Y20), traffic opening (Y21), and tourism openness (Y22). These indicators collectively capture regions’ abilities to generate new knowledge, absorb external resources, and translate innovation into coordinated economic and social development. By embedding innovation within a broader framework of openness and governance, this dimension reflects the systemic and integrative nature of innovation-driven development in the Chinese modernization context.

To operationalize the five-dimensional framework of Chinese-style modernization, this study further maps each secondary indicator (Y1–Y22) to a set of corresponding tertiary indicators, forming a structured and hierarchical evaluation system. The construction logic follows three principles: theoretical consistency, data availability, and non-redundancy.

First, each secondary indicator represents a distinct conceptual aspect within its corresponding primary dimension, ensuring consistency with the core connotations of Chinese-style modernization. For example, population quality emphasizes demographic structure and human capital, economic strength reflects the material foundation of development, social development captures inclusive and people-centered outcomes, ecological sustainability embodies green development constraints, and innovation and governance represent institutional capacity and dynamic drivers. Second, tertiary indicators are selected based on their ability to empirically operationalize each secondary indicator using officially published and continuously available provincial-level statistics. This guarantees both data reliability and temporal comparability across regions and years. Third, to reduce redundancy and potential collinearity, indicators with overlapping meanings or high conceptual similarity were screened and consolidated during the construction process. Each tertiary indicator is assigned to only one secondary indicator, following a one-to-one conceptual correspondence, thereby preserving the interpretability of the indicator system.

The resulting two–three-level indicator structure enables a clear linkage between abstract modernization concepts and measurable statistical indicators. Table 2 presents the detailed correspondence between the secondary indicators (Y1–Y22) and their associated tertiary indicators, which serves as the empirical foundation for subsequent weighting and evaluation. Table 3 presents the weights of tertiary indicators.

Within the tertiary evaluation indicators, the top five by weight include the total volume of goods import and export (0.07868), technology market turnover (0.06037), cultural manufacturing enterprises above designated size (0.05934), new product development projects (0.05668), and total foreign investment (0.05586). These indicators pertain to the “material and cultural-ethical advancement” and “peaceful development” dimensions of Chinese modernization. This suggests that regional modernization processes exhibit notable differences in peaceful development and coordination between material and spiritual civilizations, resulting in a higher dispersion of these indicators across provinces. Additionally, it underscores the preference of this paper’s comprehensive evaluation system for these two aspects, reflecting their significant contribution to evaluating and measuring Chinese modernization.

### 3.2. Evaluation Results

Utilizing the entropy-based weights and the GRM–EWT–TOPSIS framework, the comprehensive evaluation results of Chinese-style modernization for 31 provinces from 2018 to 2023 are reported in Table 4. The evaluation scores range from 0 to 1, with higher values indicating more advanced levels of modernization. Overall, the results reveal a clear hierarchical structure in regional modernization outcomes, reflecting differentiated development pathways across China.

From a spatial perspective, the highest-ranked provinces, Guangdong, Jiangsu, Zhejiang, Shandong, and Beijing are predominantly located in the eastern region. This pattern is consistent with long-standing theories of unbalanced development and coastal-led modernization in China, whereby early integration into global markets, stronger industrial foundations, and higher governance capacity have enabled eastern provinces to advance more rapidly along multiple dimensions of modernization. Guangdong stands out as the only province classified at Level I, indicating a relatively comprehensive and coordinated modernization profile across population quality, economic strength, social development, ecological sustainability, and innovation and governance.

At the same time, the classification results show that the majority of provinces fall into Level II and Level III, suggesting that Chinese modernization remains, for most regions, at an intermediate stage. This finding aligns with the notion of “stage-differentiated modernization” in large developing economies, where regions progress at varying speeds depending on their structural conditions, institutional capacity, and resource endowments [29]. Provinces in central and western China generally exhibit lower evaluation scores, reflecting persistent constraints related to industrial structure, innovation capacity, and governance effectiveness.

Importantly, the results do not indicate a simple linear gradient from east to west. Instead, considerable variation exists within each macro-region, implying that Chinese-style modernization follows multiple, non-uniform trajectories rather than a single convergence path. This heterogeneity underscores the role of localized governance models, policy implementation capacity, and regional development strategies in shaping modernization outcomes [30]. The prevalence of medium-level modernization further suggests that China’s current development phase is characterized by the coexistence of advanced, transitional, and lagging regions, highlighting the complexity and long-term nature of achieving balanced modernization nationwide.

## 4. Regional Discrepancies and Spatiotemporal Evolution Trends

### 4.1. Cluster Analysis of Evaluation Results

Cluster analysis reveals regional disparities in Chinese modernization levels based on data distribution. Utilizing the K-Means clustering algorithm, this paper categorizes the evaluation results of each province from 2018 to 2023 into clusters. The algorithm’s random number seed is set at random_state = 20, and the optimal cluster count, determined via the elbow and silhouette coefficient methods, is established at K = 4. The 31 provinces’ Chinese modernization levels are segmented into four clusters, denoted as 1, 2, 3 and 4. Furthermore, considering geographical factors, the 31 provinces are grouped into three regions: eastern, central, and western, to delve deeper into the regional distribution of the evaluation results. The clustering outcomes are displayed in Table 5.

Observations reveal that within the 11 eastern regions, notable differences in clustering results persist. The clustering results for Chinese modernization levels show that Guangdong, Shandong, Jiangsu, and Zhejiang, four coastal regions, are categorized together. This suggests that Chinese modernization levels in these four regions are relatively homogenous and differ significantly from the other 27 provinces, as evidenced by the largest inter-cluster differences in K-Means clustering. This may stem from the rapid economic development, openness, and transportation benefits of coastal areas, contributing to their enhanced resource endowments in Chinese modernization. Concurrently, Beijing, Shanghai, and Fujian are grouped into the same cluster, with Beijing and Shanghai’s modernization levels ranked in the second tier.

K-Means clustering illustrates the variance in Chinese modernization levels across regions, with regions in the same cluster exhibiting similar development stages. This study employs the Herfindahl-Hirschman Index (HHI) to further assess the intra-cluster differences, or impurity (IMP), in the level of Chinese modernization within China’s eastern, central, and western regions. Larger intra-cluster differences signify greater unevenness in Chinese modernization within those regions, hindering cross-regional collaborative cooperation and development. The formula for calculating the intra-cluster difference IMP is as follows:

(17) $$IMP\left(D\right)=1-\sum_{k=1}^{K}{\left(\frac{\left|c_{k}\right|}{C}\right)}^{2}$$

In the regional category *D*, there are K categories in the clustering results, with *c_k_* representing the samples of the *k*-th category in the dataset, k=1,2,⋯,K. The calculated intra-cluster difference results for each region are as follows:

IMP (Eastern Region) = 0.6612

IMP (Central Region) = 0.5000

IMP (Western Region) = 0.5694

This calculation result reveals that the sequence of intra-cluster variability is: Eastern Region > Western Region > Central Region. This suggests that Chinese modernization in the Central Region’s eight provinces is comparatively uniform and stable, whereas the Eastern Region exhibits more diversity and uncertainty in its modernization, exceeding the disparities observed in the Central and Western Regions.

### 4.2. Kernel Density Estimation

**Figure 3:**
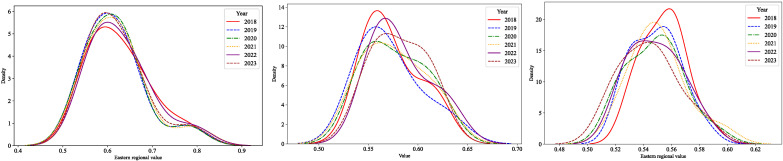
Kernel density estimation for diverse regions.

This study utilizes kernel density Estimation (KDE) to construct probability density curves for the levels of Chinese modernization in the eastern, central, and western regions, analyzing the spatiotemporal evolution from 2018 to 2023. KDE, which utilizes kernel functions to estimate probability density, operates without the need for prior data knowledge or assumptions about data distribution, allowing for direct analysis of the distribution. The Epanechnikov function has been selected as the kernel function in this analysis. The results of the kernel density estimation for these regions are depicted in Figure 3.

To examine the dynamic evolution and internal distribution of Chinese-style modernization across regions, KDE is employed to analyze the temporal changes in modernization levels in the eastern, central, and western regions from 2018 to 2023. Using the Epanechnikov kernel function, KDE allows for a non-parametric assessment of distributional shifts without imposing prior assumptions on data structure, thereby capturing subtle changes in regional disparity over time.

The kernel density curves for the eastern region exhibit noticeable horizontal fluctuations accompanied by a gradual widening of the distribution. This pattern indicates that while the overall level of modernization remains relatively high, internal differentiation has intensified over time. From a theoretical perspective, this reflects the transition of advanced regions from extensive growth toward quality-oriented development, where disparities increasingly stem from differences in innovation efficiency, governance capacity, and ecological constraints rather than from basic economic factors alone. In the central region, the kernel density curves display a clear rightward shift with a moderate increase in width, suggesting an overall improvement in modernization levels. Notably, the narrower peak observed around 2021 implies a temporary reduction in internal disparity. This evolution is consistent with a catch-up or transitional modernization pathway, in which structural upgrading, industrial relocation, and improved connectivity contribute to gradual convergence among provinces.

By contrast, the western region demonstrates a distinct trajectory. The kernel density peaks shift leftward over time, accompanied by an expanding distribution width, indicating both declining average modernization levels and growing internal imbalance. This pattern reflects persistent structural constraints faced by western provinces, including limited innovation capacity, fragile ecological conditions, and uneven governance effectiveness. Rather than converging toward more advanced regions, the western region exhibits signs of divergence, highlighting the challenges of achieving balanced modernization under heterogeneous development conditions.

Building on the analysis of the measurement and dynamic evolution of the level of Chinese modernization in various provincial regions, a time series convergence model is employed to further dissect the convergence characteristics of modernization development in different areas. The σ convergence is particularly effective in reflecting the temporal changes in differences between regions and in depicting the stock variable of regional evolutionary differences. Therefore, the σ convergence values for the eastern, central, and western regions are calculated, with the basic formula being:

(18) $$\sigma_{t}=\frac{\sqrt{\frac{1}{n}\sum_{i=1}^{n}{\left(CM_{it}-C{\bar{M}}_{t}\right)}^{2}}}{C{\bar{M}}_{t}}$$

**Figure 4:**
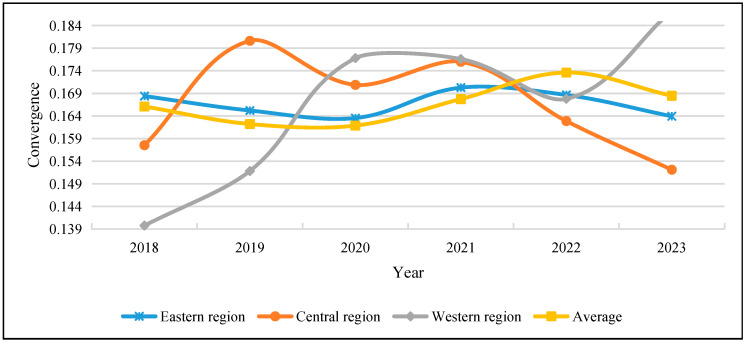
Convergence trends of Chinese modernization for diverse regions.

Here, $CM_{it}$ represents the evaluation result of Chinese modernization for region *i* in year *t*, and is the comprehensive evaluation result for year *t*. Based on Equation (18), the σ convergence values for China’s eastern, central, and western regions are calculated, as shown in Figure 4.

The regional convergence trends reveal that the nationwide evolution of Chinese-style modernization exhibits an approximate inverted U-shaped temporal pattern. Specifically, regional disparities initially narrowed, subsequently expanded, and then showed signs of partial adjustment. This non-linear convergence trajectory suggests that modernization in China does not follow a smooth or monotonic convergence path, but instead reflects stage-dependent dynamics shaped by shifting development priorities and structural conditions. The eastern region demonstrates a convergence pattern broadly consistent with the national trend. In the early stages, disparities narrowed as advanced provinces leveraged economic scale and openness to consolidate development gains. However, as modernization progressed toward higher-quality dimensions, such as innovation efficiency, ecological sustainability, and governance effectiveness, internal differences re-emerged, indicating that convergence based on factor accumulation alone has reached its limits. In contrast, the central region exhibits a gradual reduction in dispersion, suggesting a relative convergence process. Although full convergence has not yet been achieved, the declining convergence interval indicates that differences in modernization levels are becoming smaller over time. This pattern is consistent with a transitional catch-up trajectory, where industrial upgrading, infrastructure connectivity, and policy coordination facilitate incremental narrowing of regional gaps. The western region, however, displays a clear divergence trend, with widening disparities over time. This divergence reflects persistent structural constraints, including limited innovation capacity, ecological vulnerability, and uneven governance effectiveness. Rather than converging toward more advanced regions, some western provinces appear to be trapped in a development lock-in, underscoring the difficulty of achieving uniform modernization under heterogeneous initial conditions.

Overall, the convergence analysis reinforces the view that Chinese-style modernization is characterized by differentiated and non-linear regional evolution. The coexistence of partial convergence, stagnation, and divergence across regions highlights the necessity of region-specific policy interventions and governance strategies. These findings further suggest that achieving balanced modernization in China requires addressing not only economic gaps but also institutional and structural disparities that shape long-term regional development trajectories.

## 5. Conclusions and Suggestions

### 5.1. Conclusions

This study develops a comprehensive evaluation framework to assess regional Chinese-style modernization, integrating grey relational modeling, entropy-based weighting, and the TOPSIS method. By operationalizing modernization through five theoretically grounded dimensions, population quality, economic strength, social development, ecological sustainability, and innovation and governance, the framework captures both the multidimensional nature and the spatial heterogeneity of China’s modernization process. Applying this approach to provincial data from 2018 to 2023 enables a systematic examination of modernization levels, regional disparities, and dynamic evolution patterns across China.

The empirical results indicate that, overall, most provinces remain at a moderate stage of modernization, while pronounced spatial imbalances persist. Eastern provinces generally exhibit higher modernization levels, reflecting stronger economic foundations, greater openness, and more concentrated innovation resources. However, cluster analysis reveals substantial intra-regional heterogeneity within the eastern region, suggesting that high average performance coexists with notable internal volatility. In contrast, the central region demonstrates relatively homogeneous modernization levels, with evidence of gradual convergence, whereas the western region is characterized by persistent low performance and increasing internal disparities.

Further spatiotemporal analysis reinforces these findings. Kernel density estimation shows divergent regional trajectories: the eastern region displays widening internal differentiation, the central region experiences a rightward shift accompanied by modest dispersion reduction, and the western region exhibits both declining overall modernization levels and growing imbalance. Convergence analysis reveals an approximate inverted U-shaped national trend, indicating that regional disparities initially narrowed, then expanded, and have recently shown signs of partial adjustment. Importantly, convergence dynamics differ markedly across regions, with the central region moving toward relative convergence and the western region exhibiting clear divergence.

Taken together, these results highlight that Chinese-style modernization is not a uniform or linear process but one shaped by region-specific structural conditions, development stages, and governance capacities. The findings underscore the necessity of differentiated policy strategies that move beyond one-size-fits-all approaches. Strengthening innovation capacity and governance effectiveness in lagging regions, improving ecological sustainability, and addressing internal disparities within advanced regions are essential for promoting more balanced, inclusive, and sustainable modernization. The proposed evaluation framework, thus, provides a transparent and adaptable analytical tool to support evidence-based regional policy design and long-term monitoring of China’s modernization trajectory.

### 5.2. Policy Iomplications

The empirical findings of this study carry several important policy implications for advancing Chinese-style modernization in a more balanced and sustainable manner. First, the pronounced inter- and intra-regional disparities revealed by the analysis indicate that a uniform modernization strategy is unlikely to be effective. Policymakers should adopt differentiated and region-specific approaches that align with local development stages, resource endowments, and institutional capacities.

For eastern provinces, which generally exhibit higher modernization levels but greater internal volatility, policy priorities should shift from scale expansion toward quality enhancement. Emphasis should be placed on strengthening innovation governance, reducing internal inequality, and improving ecological efficiency to mitigate the risks associated with uneven development within advanced regions. Coordinating economic growth with environmental sustainability and social inclusiveness remains crucial for maintaining long-term modernization momentum.

In the central region, where modernization levels are relatively homogeneous and convergence trends are emerging, policies should focus on consolidating these gains. Enhancing regional coordination, upgrading industrial structures, and improving public service provision can help transform gradual convergence into sustained and stable advancement. Strengthening inter-provincial cooperation may further facilitate the diffusion of development benefits within the region.

The western region faces the most pressing challenges, characterized by persistent low modernization levels and increasing internal divergence. Targeted policy interventions are, therefore, essential. Priority should be given to improving basic innovation capacity, governance effectiveness, and infrastructure connectivity, alongside ecological protection measures tailored to fragile environments. Strengthening institutional support and fostering cross-regional collaboration can help address structural constraints and prevent further divergence.

More broadly, the results underscore the importance of integrating population quality, innovation capacity, and governance effectiveness into modernization policy frameworks. By using the proposed evaluation system as a monitoring and diagnostic tool, policymakers can more accurately identify regional bottlenecks, track dynamic changes, and design adaptive strategies that promote inclusive, coordinated, and sustainable modernization nationwide.

### 5.3. Limitations and Future Directions

Despite its contributions, this study has several limitations that should be acknowledged and addressed in future research. First, although the indicator system is designed to capture key dimensions of Chinese-style modernization, data availability constraints necessitated reliance on officially published statistics. Some qualitative aspects of governance capacity, social cohesion, and institutional quality may, therefore, be underrepresented. Future studies could incorporate survey-based indicators or alternative data sources to enrich the measurement framework.

Second, while the GRM–EWT–TOPSIS framework effectively captures relative modernization levels and spatial disparities, it does not explicitly model causal mechanisms. Future work could integrate econometric or spatial regression techniques to explore the drivers of regional modernization differences and assess the impacts of specific policy interventions.

## Figures and Tables

**Table 1:** Evaluation results scale.

**Evaluation Value**	≤0.35	(0.35, 0.60]	(0.60, 0.75]	>0.75
**Grade**	IV	III	II	I

**Table 2:** Mapping of secondary and tertiary indicators.

Symbol	Indicator	Symbol	Indicator	Symbol	Indicator
Y1-x1	Total population	Y8-x17	Gap between urban and rural income levels	Y15-x32	Per capita electricity consumption
Y1-x2	Share of urban population at the end of the year	Y8-x18	Urban unemployment rate	Y15-x33	Per capita water consumption
Y2-x3	Ratio of male to female population	Y9-x19	Per capita food consumption expenditure	Y16-x34	Sulfur dioxide emissions
Y2-x4	Urban population density	Y9-x20	Per capita consumption expenditure on recreation	Y16-x35	Nitrogen oxide emissions
Y3-x5	Share of population aged 0 to 14 years	Y10-x21	Investment in education	Y17-x36	Forest fires
Y3-x6	Share of 15–64 year olds	Y10-x22	Teacher-student ratio in higher education	Y17-x37	Geologic hazards
Y3-x7	Proportion of persons aged 65 and over	Y11-x23	Number of cultural manufacturing enterprises above scale	Y18-x38	Domestic waste removal
Y4-x8	Illiterate population aged 15 and over	Y11-x24	Artistic performances	Y18-x39	Investment in industrial pollution control
Y4-x9	Students in higher education per 100,000 population	Y11-x25	Combined population coverage of radio programs	Y19-x40	Total exports and imports of goods
Y5-x10	Gross regional product (GDP)	Y12-x26	Number of patents granted	Y19-x41	Total foreign investment
Y5-x11	consumer price index CPI	Y12-x27	Technology market turnover	Y19-x42	Share of e-commerce transactions
Y6-x12	GDP per capita growth rate	Y13-x28	Public library holdings per capita	Y20-x43	Internet broadband subscriber accesses
Y6-x13	Growth rate of investment in fixed assets	Y13-x29	Number of new product development projects	Y20-x44	Websites per 100 businesses
Y7-x14	Number of persons covered by health insurance	Y14-x30	forest cover	Y21-x45	Railroad mileage
Y7-x15	Number of persons insured for old-age pension	Y14-x31	Land use area	Y21-x46	Regional freight traffic
Y7-x16	Number of persons insured against work-related injuries			Y21-x47	Vehicle ownership for road operations
				Y22-x48	Passenger turnover

**Table 3:** Calculation of the weights of tertiary indicators.

Indicator	2018	2019	2020	2021	2022	2023	Combined Weights
x1	0.00852	0.00687	0.00672	0.00687	0.00679	0.00663	0.00707
x2	0.00737	0.00736	0.00680	0.00669	0.00650	0.00623	0.00682
x3	0.00651	0.00868	0.00615	0.00415	0.00650	0.00760	0.00660
x4	0.00905	0.00726	0.00871	0.00941	0.00928	0.01102	0.00912
x5	0.02306	0.02293	0.02358	0.02519	0.02633	0.02618	0.02454
x6	0.01733	0.01720	0.01712	0.01797	0.01947	0.01934	0.01807
x7	0.01081	0.00879	0.00675	0.00716	0.01010	0.00981	0.00891
x8	0.00838	0.00258	0.00264	0.00280	0.00280	0.00261	0.00364
x9	0.01133	0.01225	0.01185	0.01130	0.00989	0.01042	0.01117
x10	0.02256	0.02296	0.02316	0.02511	0.02544	0.02491	0.02402
x11	0.00543	0.00480	0.02152	0.00813	0.00653	0.01348	0.00998
x12	0.00286	0.00789	0.00900	0.00830	0.00403	0.01473	0.00780
x13	0.00257	0.00374	0.00634	0.00608	0.00420	0.00453	0.00458
x14	0.03070	0.02307	0.01941	0.02026	0.02032	0.02048	0.02237
x15	0.02626	0.02570	0.02560	0.02630	0.02596	0.02541	0.02587
x16	0.02585	0.02612	0.02629	0.02758	0.02787	0.02826	0.02700
x17	0.00445	0.00431	0.00430	0.00441	0.00467	0.00470	0.00447
x18	0.01980	0.01452	0.01507	0.00888	0.00879	0.00921	0.01271
x19	0.02046	0.02200	0.02650	0.02650	0.02765	0.02064	0.02396
x20	0.00766	0.00777	0.00818	0.00845	0.00597	0.00687	0.00748
x21	0.01903	0.01849	0.02047	0.02139	0.02250	0.02336	0.02087
x22	0.01497	0.01252	0.00709	0.00676	0.00593	0.01022	0.00958
x23	0.05691	0.05702	0.05629	0.06171	0.06242	0.06172	0.05934
x24	0.06400	0.06254	0.05456	0.04055	0.05297	0.04992	0.05409
x25	0.00504	0.00348	0.00313	0.00313	0.00294	0.00284	0.00343
x26	0.04531	0.04664	0.04787	0.04975	0.04953	0.04670	0.04763
x27	0.07300	0.06733	0.05704	0.05865	0.05667	0.04953	0.06037
x28	0.02114	0.03160	0.03257	0.03229	0.03408	0.02982	0.03025
x29	0.05333	0.05557	0.05683	0.05913	0.05805	0.05714	0.05668
x30	0.01690	0.01671	0.01645	0.01710	0.01717	0.01695	0.01688
x31	0.03166	0.03133	0.03150	0.03276	0.02832	0.02795	0.03059
x32	0.00560	0.00592	0.00585	0.00726	0.00587	0.00745	0.00633
x33	0.00288	0.00290	0.00294	0.00298	0.00310	0.00307	0.00298
x34	0.00488	0.00829	0.00833	0.01247	0.00677	0.00779	0.00809
x35	0.00698	0.00656	0.00659	0.00682	0.00305	0.00820	0.00637
x36	0.00377	0.00400	0.00325	0.00484	0.00390	0.00610	0.00431
x37	0.00273	0.00251	0.00482	0.00276	0.00343	0.00548	0.00362
x38	0.02118	0.02199	0.02287	0.02500	0.02409	0.02374	0.02315
x39	0.02862	0.03075	0.03092	0.03326	0.03444	0.02543	0.03057
x40	0.08211	0.07847	0.07730	0.07953	0.07837	0.07630	0.07868
x41	0.05314	0.05885	0.05646	0.05319	0.05471	0.05882	0.05586
x42	0.01478	0.01641	0.01805	0.01790	0.01986	0.01930	0.01772
x43	0.02290	0.02229	0.02066	0.02051	0.02017	0.01959	0.02102
x44	0.00423	0.00504	0.00568	0.00894	0.00750	0.00895	0.00673
x45	0.01610	0.01590	0.01580	0.01601	0.01629	0.01520	0.01588
x46	0.01795	0.01828	0.01832	0.01943	0.01975	0.01864	0.01873
x47	0.02056	0.02177	0.02210	0.02285	0.02635	0.02571	0.02322
x48	0.01932	0.01999	0.02056	0.02150	0.02270	0.02103	0.02085

**Table 4:** Evaluation results and ranking of the level of Chinese modernization in each province and region.

Province	2018	2019	2020	2021	2022	2023	Average Value	Rank	Label
Beijing	0.6449	0.6289	0.6265	0.6327	0.6235	0.6150	0.6286	5	II
Tianjin	0.5602	0.5501	0.5479	0.5572	0.5570	0.5547	0.5545	20	III
Hebei	0.5869	0.5820	0.5824	0.5893	0.5977	0.5850	0.5872	9	III
Shanxi	0.5446	0.5412	0.5505	0.5502	0.5540	0.5437	0.5474	25	III
Inner Mongolia	0.5657	0.5545	0.5531	0.5516	0.5471	0.5520	0.5540	22	III
Liaoning	0.5646	0.5634	0.5632	0.5628	0.5635	0.5578	0.5625	16	III
Jilin	0.5520	0.5446	0.5467	0.5400	0.5565	0.5564	0.5494	24	III
Heilongjiang	0.5535	0.5479	0.5449	0.5457	0.5596	0.5630	0.5524	23	III
Shanghai	0.6139	0.6124	0.6222	0.6232	0.6227	0.6178	0.6187	7	II
Jiangsu	0.7040	0.6672	0.6615	0.6732	0.6972	0.6777	0.6801	2	II
Zhejiang	0.6695	0.6380	0.6408	0.6449	0.6653	0.6449	0.6506	3	II
Anhui	0.6103	0.6027	0.6118	0.6146	0.6179	0.6193	0.6128	8	II
Fujian	0.5877	0.5851	0.5911	0.5865	0.5854	0.5785	0.5857	12	III
Jiangxi	0.5610	0.5609	0.5661	0.5696	0.5716	0.5715	0.5668	15	III
Shandong	0.6431	0.6382	0.6412	0.6372	0.6594	0.6568	0.6460	4	II
Henan	0.6181	0.6317	0.6216	0.6220	0.6311	0.6094	0.6223	6	II
Hubei	0.5816	0.5745	0.5948	0.5869	0.5774	0.5999	0.5859	11	III
Hunan	0.5683	0.5736	0.5825	0.5809	0.5902	0.5858	0.5802	13	III
Guangdong	0.7762	0.7799	0.7773	0.7916	0.7894	0.7826	0.7828	1	I
Guangxi	0.5584	0.5527	0.5514	0.5516	0.5573	0.5559	0.5546	19	III
Hainan	0.5517	0.5466	0.5438	0.5453	0.5705	0.5764	0.5557	18	III
Chongqing	0.5631	0.5663	0.5648	0.5734	0.5706	0.5729	0.5685	14	III
Sichuan	0.5855	0.5809	0.5886	0.5949	0.5831	0.5840	0.5862	10	III
Guizhou	0.5424	0.5399	0.5444	0.5429	0.5378	0.5437	0.5419	26	III
Yunnan	0.5564	0.5575	0.5584	0.5569	0.5481	0.5473	0.5541	21	III
Tibet	0.5591	0.5512	0.5511	0.5488	0.5240	0.5151	0.5415	27	III
Shanxi	0.5676	0.5619	0.5636	0.5592	0.5628	0.5508	0.5610	17	III
Gansu	0.5470	0.5310	0.5311	0.5412	0.5601	0.5367	0.5412	28	III
Qinghai	0.5418	0.5338	0.5254	0.5333	0.5240	0.5268	0.5309	29	III
Ningxia	0.5306	0.5280	0.5218	0.5208	0.5320	0.5207	0.5256	31	III
Xinjiang	0.5334	0.5267	0.5258	0.5306	0.5344	0.5256	0.5294	30	III

**Table 5:** K-Means clustering results and area division.

Province	District	Grading	Clustering Results	Provinces	District	Grading	Clustering Results
Beijing	eastern	II	2	Henan	central	II	2
Tianjin	eastern	III	4	Hubei	central	III	2
Hebei	eastern	III	2	Hunan	central	III	2
Liaoning	eastern	III	4	Inner Mongolia	western	III	4
Shanghai	eastern	II	2	Guangxi	western	III	4
Jiangsu	eastern	II	3	Chongqing	western	III	4
Zhejiang	eastern	II	3	Sichuan	western	III	2
Fujian	eastern	III	2	Guizhou	western	III	1
Shandong	eastern	II	3	Yunnan	western	III	4
Guangdong	eastern	I	3	Tibet	western	III	1
Hainan	eastern	III	4	Shanxi	western	III	4
Shanxi	central	III	4	Gansu	western	III	1
Jilin	central	III	4	Qinghai	western	III	1
Heilongjiang	central	III	4	Ningxia	western	III	1
Anhui	central	II	2	Xinjiang	western	III	1
Jiangxi	central	III	4				

## Data Availability

Data in this study are available from the corresponding author upon reasonable request.
